# Phase I dose-escalation study of F14512, a polyamine-vectorized topoisomerase II inhibitor, in patients with platinum-refractory or resistant ovarian cancer

**DOI:** 10.1007/s10637-018-0688-4

**Published:** 2018-12-14

**Authors:** Alexandra Leary, Christophe Le Tourneau, Andrea Varga, Marie-Paule Sablin, Carlos Gomez-Roca, Nicolas Guilbaud, Aurelie Petain, Mariya Pavlyuk, Jean-Pierre Delord

**Affiliations:** 1 Gustave Roussy, Oncology Department, Université Paris-Saclay, F-94805 Villejuif, France; 2 INSERM U981, Villejuif, France; 3 Department of Medical Oncology, Paris & Saint-Cloud, Institut Curie, Paris, France; 4 INSERM U900 Research unit, Saint-Cloud, France; 5 Gustave Roussy Cancer Campus, Drug Development Department, Villejuif, France; 6 Institut Claudius Regaud, IUCT-Oncopole, Departement d’Oncologie Medicale, Toulouse, France; 7 Institut de Recherche Pierre Fabre, Toulouse, France

**Keywords:** Ovarian cancer, Dose-escalation, F14512, Pharmacokinetics, Safety, Efficacy

## Abstract

*Purpose* To determine the maximum tolerated dose (MTD) of F14512, a topoisomerase II inhibitor designed to target cancer cells through the polyamine transport system, (three-hour daily infusion given for 3 consecutive days every 3 weeks) in platinum-refractory or resistant ovarian cancer. Other objectives were safety, pharmacokinetics (PK), PK/pharmacodynamics relationship, and efficacy. *Methods* This was an open-label, dose-escalation, multicenter phase I study. *Results* Eleven patients were enrolled and were treated at dose levels (DLs) of 10 and 5 mg/m^2^/day. All patients received the 3 injections per cycle as per study protocol (median, 1 cycle (Ferlay et al. Int J Cancer 136:E359–386, 2015; Siegel et al. CA Cancer J Clin 65:5–29, 2015; Oronsky et al. Med Oncol 34:103, 2017; Barret et al. Cancer Res 68:9845–9853, 2008; Ballot et al. Apoptosis 17:364–376, 2012; Brel et al. Biochem Pharmacol 82:1843–1852, 2011; Gentry et al. Biochemistry 50:3240–3249, 2011; Kruczynski et al. Investig New Drugs 29:9–21, 2011; Chelouah et al. PLoS One 6:e23597, 2011)) with no dose reductions. At DL 10 mg/m^2^/day, 6 dose-limiting toxicities (DLTs) were reported (3/4 evaluable patients: 2 grade 3 febrile neutropenia, 1 grade 4 neutropenia lasting at least 7 days, 1 grade 3 nausea, 1 decreased appetite, and 1 grade 3 asthenia). At dose 5 mg/m^2^/day, 2 DLTs were reported (2/6 treated patients: 2 grade 3 febrile neutropenia). Both DLs were defined as MTD. Stable disease was reported as best overall response in 2 (40%) patients having both received 9 cycles, one at each DL. 90.9% of patients experienced grade 4 neutropenia, but for only one (9.1%) it was reported as a serious adverse event. *Conclusion* Although there was some encouraging efficacy signal, grade 4 neutropenia led to complications and it was decided to stop the study. A DL below 5 mg/m^2^/day was not tested as this would not allow reaching the minimum serum concentration needed for the pharmacological activity of the drug.

**Electronic supplementary material:**

The online version of this article (10.1007/s10637-018-0688-4) contains supplementary material, which is available to authorized users.

## Introduction

Ovarian cancer is the seventh most common malignancy and the second most common gynecological malignancy, accounting for approximately 239,000 newly diagnosed cases and 152,00 deaths worldwide [1]. In 2017, the number of newly diagnosed cases in the USA is estimated to be more than 22,000 and the number of deaths was expected to exceed 14,000 [2]. In Europe in 2012, an estimated 66,000 new cases of ovarian cancer were diagnosed, resulting in approximately 42,000 deaths [1]. The current standard of care for newly diagnosed ovarian cancer is debulking surgery, followed by carboplatin and paclitaxel chemotherapy. Despite approximately 80% response rates to primary therapy, over half of the patients relapse within 2 years and eventually develop platinum-resistant disease. Treatment options for platinum-resistant ovarian cancer (PROC) are clearly unsatisfactory, with response rates to paclitaxel or pegylated liposomal doxorubicin (PLD) ranging from 0%–30% and progression free survival of 2–6 months. [3]. Thus, there is an unmet clinical need to identify new therapeutic options for patients with PROC.

Vectorization can improve tumor selectivity of conventional cytotoxics by conjugating them with a chemical entity to target cancer cells more specifically. One strategy is to exploit a selective transport system, such as the polyamine transport system (PTS) which is overactive in many tumor cells. The anticancer drug candidate F14512 was designed to target cancer cells through the PTS. It contains a spermine chain in place of the C4 glyosidic moiety of etoposide. The positively charged spermine tail increases DNA binding to reinforce topoisomerase II (topo II) inhibition and favors the selective uptake of the drug by tumor cells via the PTS, resulting in decreased toxicity when used in vivo [4–11]. In vitro, the superior anti-proliferative activity of F14512 was demonstrated in 21/29 human cancer cell lines [4]. In vivo, F14512 demonstrated antitumor efficacy in 13/19 experimental models used, yielding a response rate of 68%. In these models, complete tumor regression was observed after intravenous or oral administration of F14512 and antitumor activity was observed over a range of dose levels (DLs) providing evidence of its good tolerance [8].

The clinical efficacy of topo II inhibitors has been established in ovarian cancer [12, 13]. PTS activity in ovarian tumors was investigated and the differential uptake of F17073, a polyamine fluorescent probe acting as a biomarker of PTS activity, by ovarian cancer versus normal cells was demonstrated in 14/17 evaluable clinical samples analyzed ex vivo [14]. Preclinical studies using the SK-OV3 ovarian cancer cell line confirmed the high levels of PTS activity previously observed in patient samples. Indeed, a ten-fold increased sensitivity of SK-OV3 cancer cells to F14512 cytotoxicity compared to etoposide, a non-targeted topo II inhibitor, was observed [14]. Preliminary studies, which were performed in ovarian tumor models, such as the PTS(+) A2780R cisplatin-resistant cell line, confirmed the antitumor activity of F14512 and its potential as a new therapy for PROC [14].

The primary objective was to determine the maximum tolerated dose (MTD) of F14512 administered as a three-hour daily infusion given for 3 consecutive days (on days 1, 2, 3) every 3 weeks in women with platinum-refractory or resistant ovarian cancer. The secondary objectives were i) to assess the safety of F14512, ii) to characterize the pharmacokinetics (PK) and the PK/ pharmacodynamics (PD) relationship of F14512 and its metabolite F16490, iii) to assess the efficacy of F14512 according to Gynecologic Cancer Intergroup (GCIG) response criteria [15].

## Methods

### Patients and study design

Patients with histologically or cytologically confirmed advanced epithelial ovarian cancer, fallopian tube or peritoneal carcinoma considered platinum refractory or resistant, with no more than 2 prior platinum-based regimens, were eligible. Platinum-resistant disease was defined as progressive disease within 6 months of completing the most recent platinum-based regimen. Patient eligibility criteria are described in the Online Resource.

This was an open-label, dose-escalation, multicenter, phase I study (EudraCT Number: 2012–005798-29) conducted in 3 investigational sites in France. The 10 mg/m^2^/day dose of F14512 was planned as a starting dose (DL1) for escalation in cohorts of 3 to 6 patients until the MTD was reached. Dose escalation was based on a standard titration design by using increments of 33% except from the first dose escalation, which could proceed with a dose increment of 50% in the absence of grade 3 neutropenia and dose limiting toxicity (DLT) observed at DL1. Dose escalation then proceeded except if criteria of MTD were met at DL1. In that instance, the 5 mg/m^2^/day dose was tested. All patients in a DL had to be followed for at least 3 weeks (cycle 1) for DLT evaluation before the first patient of the next dose level started treatment. The final decision to proceed to the next DL was made by the safety committee at the dose-escalation meeting. The MTD was defined as the DL at which 2/3 or 2/6 patients experienced a DLT during the first cycle. The Recommended Dose (RD) was the DL immediately below the MTD. A total of 10 evaluable patients had to be treated at the RD.

Treatment could be continued until disease progression, unacceptable toxicity, patient’s request to discontinue treatment or intercurrent illness which required treatment discontinuation in the investigator’s opinion.

No routine premedication was recommended. However, magnesium supplementation could be needed from the first day of treatment with F14512 depending on serum magnesium values during the cycle.

F14512 was administered as three-hour daily infusion through a central venous catheter for 3 consecutive days every 3 weeks depending on recovery to normal hematopoiesis or recovery of non-hematological toxicities to grade 1. During the first cycle, G- and GM-CSF use was prohibited, however, there were no such constraints in subsequent cycles.

### Criteria for evaluation

Toxicities were graded according to National Cancer Institute - Common Toxicity Criteria (NCI - CTC) for adverse events (AEs) (version 4.0). DLT determination criteria and safety assessments are described in the Online Resource. Response was assessed by using GCIG response criteria [15] incorporating RECIST version 1.1 and CA-125. Plasma PK assessments and statistical methods are described in the Online Resource.

## Results

### Patient characteristics

A total of 11 patients were enrolled in the study and all were treated between June 2013 and May 2014. The patient baseline characteristics and demographics were comparable between the 2 DLs and are summarized in Table 1. Overall, patient median age was 63 (57.7–69.9) years while performance status was 0 in 36.4% of patients and 1 in 63.6% of patients. Median progression free interval was 0.4 months (0.0–7.7) and 36.4% of patients had ≥3 organs involved at baseline.

**Table 1:** Baseline patient and disease characteristics

Characteristic	Dose level of F14512		
	10 mg/m^2^/day (*n* = 5) N (%)	5 mg/m^2^/day (*n* = 6) N (%)	Overall (*n* = 11) N (%)
Age (years)			
Median	63.1	62.2	63.0
Range	[61.4–69.9]	[57.7–68.9]	[57.7–69.9]
Groups of age, N (%)			
< 65	3 (60.0)	4 (66.7)	7 (63.6)
≥ 65	2 (40.0)	2 (33.3)	4 (36.4)
WHO performance status at baseline, N (%)			
0	2 (40.0)	2 (33.3)	4 (36.4)
1	3 (60.0)	4 (66.7)	7 (63.6)
Body weight (kg)			
Median	65.0	63.7	65.0
Range	[49.7–83.0]	[50.0–86.9]	[49.7–86.9]
BSA (m^2^)			
Median	1.7	1.7	1.7
Range	[1.5–1.8]	[1.5–2.0]	[1.5–2.0]
Primary tumor site			
Epithelial ovarian cancer	4 (80.0)	4 (66.7)	8 (72.7)
Peritoneal carcinoma	1 (20.0)	2 (33.3)	3 (27.3)
Histology, N (%)			
Serous Carcinoma	4 (80.0)	4 (66.7)	8 (72.7)
Endometroid carcinoma	1 (20.0)	1 (16.7)	2 (18.2)
Unknown	–	1 (16.7)	1 (9.1)
Histopathological grade, N (%)			
G2 moderately differentiated	2 (40.0)	–	2 (18.2)
G2 poorly differentiated	2 (40.0)	4 (66.7)	6 (54.5)
Unknown	1 (20.0)	2 (33.3)	3 (27.3)
Figo stage, N (%)			
III B	1 (20.0)	1 (16.7)	2 (18.2)
III C	–	3 (50.0)	3 (27.3)
III	2 (40.0)	–	2 (18.2)
IV	–	2 (33.3)	2 (18.2)
IC	1 (20.0)	–	1 (9.1)
Unknown	1 (20.0)	–	1 (9.1)
Time from first diagnosis to study entry (months)			
Median	28.1	16.2	18.6
[Range]	[11.2–44.8]	[9.7–25.4]	[9.7–44.8]
Progression free interval (months)			
Median	0.4	0.4	0.4
[Range]	[0.0–7.7]	[0.0–0.7]	[0.0–7.7]
Unknown	1	1	2
Number of organs involved, N (%)			
1 organ	1 (20.0)	2 (33.3)	3 (27.3)
2 organs	1 (20.0)	3 (50.0)	4 (36.4)
≥ 3 organs	3 (60.0)	1 (16.7)	4 (36.4)
Type of organs involved at baseline, N (%)			
Liver	3 (60.0)	5 (83.3)	8 (72.7)
Lymph nodes	3 (60.0)	2 (33.3)	5 (45.5)
Peritoneum	1 (20.0)	2 (33.3)	3 (27.3)
Colon	1 (20.0)	1 (16.7)	2 (18.2)
Pleural effusion	2 (40.0)	–	2 (18.2)
Other	2 (40.0)	1 (16.7)	3 (27.3)

*BSA* body surface area, *WHO* World Health Organization

### Determination of MTD and DLTs

Five and 6 patients were treated with 10 mg/m^2^/day and 5 mg/m^2^/day, respectively, administered for 3 consecutive days, every 3 weeks. Patients received a median number of 1 cycle [1–9] and 1.5 cycles [1–9] at DL 10 mg/m^2^/day and DL 5 10 mg/m^2^/day, respectively. At DL 10 mg/m^2^/day, 6 DLTs were reported in 3/4 evaluable patients for MTD determination (Table 2). One patient was not evaluable because of incomplete cycle 1 due to a fatal unrelated AE (ischemic stroke). DLTs were hematological toxicities for 3 patients; 2 grade 3 febrile neutropenia and 1 grade 4 neutropenia lasting at least 7 days. Gastrointestinal toxicities of grade 3, which were DLTs, were reported in 1/3 patients (nausea, decreased appetite associated with grade 3 asthenia). Therefore, as per study protocol, dose was de-escalated to 5 mg/m^2^/day. At dose 5 mg/m^2^/day, 2 DLTs were reported in 2/6 treated patients. They were both hematological toxicities; 2 grade 3 febrile neutropenia. Permanent treatment discontinuation because of study drug-related toxicity was observed only at cycle 1 in 5 patients. All discontinuations were because of DLTs; 3 in patients treated at DL 10 mg/m^2^/day and 2 in patients treated at DL 5 mg/m^2^/day.

**Table 2:** DLTs occuring in the dose-escalation phase

Dose of F14512 (mg/m^2^/day)	Treated/evaluable patients (N)	Patients with DLT (N)	Total cycles N (%)	Cycles administered median [range]	Type of DLTs (N)
10	5/4	3	13 (100.0)	1.0 [1.0–9.0]	Grade 3 Febrile neutropenia (2) Grade 3 Nausea (1) Grade 3 Asthenia (1) Grade 3 Decreased appetite (1) Grade 4 Neutropenia lasting at least 7 days (1)
5	6/6	2	16 (83.3)	1.5 [1.0–9.0]	Grade 3 Febrile neutropenia (2)

Both DLs were defined as MTD because of DLTs observed.

A total of 29 cycles were administered, 13 at DL 10 mg/m^2^/day and 16 at DL 5 mg/m^2^/day. The first 3 patients received 10 mg/m^2^/day for 3 consecutive days. All patients received the 3 injections per cycle as planned by the study protocol. No dose reductions were reported.

### Safety

All 11 patients were evaluable for safety. Hematological toxicities were mainly grade 4 neutropenia. They were experienced by 90.9% of treated patients and observed in 26/29 cycles administered. This neutropenia was reported as a serious drug-related AE in 1 patient (9.1%) only. Grade 3 febrile neutropenia was observed in 4 patients (36%), 2 at each DL. Neutropenic infection was observed in 1 patient at DL 5 mg/m^2^/day. Main non-hematological toxicities were general disorders reported in 9 patients (82%), experiencing mainly asthenia. Gastrointestinal disorders were reported in 6 patients (55%); nausea in 6 patients (55%) and vomiting in 4 patients (36%). Metabolism and nutrition disorder with decreased appetite in 5 patients (46%). Study drug-related AEs are summarized in Table 3. Study drug-related serious AEs (SAEs) were reported in 6 patients (55%); 3 patients at each DL and were hematological toxicities. Five of these 6 SAEs were DLTs; grade 3 febrile neutropenia was reported in 2 patients at each DL and grade 4 neutropenia lasting at least 7 days was reported in 1 patient at DL 10 mg/m^2^/day. There were no study drug-related deaths.

**Table 3:** Grade 3–4 study drug-related adverse events (worse grade by patient)

		Dose level of F14512								
System Organ Class	Preferred Term	10 mg/m^2^/day n = 5			5 mg/m^2^/day n = 6			Overall n = 11		
		Grade 3 N (%)	Grade 4 N (%)	Overall N (%)	Grade 3 N (%)	Grade 4 N (%)	Overall N (%)	Grade 3 N (%)	Grade 4 N (%)	Overall N (%)
Any related TESS		2 (40.0)	1 (20.0)	5 (100.0)	4 (66.7)	0 (0.0)	6 (100.0)	6 (54.5)	1 (9.1)	11 (100.0)
Blood and lymphatic system disorders		2 (40.0)	1 (20.0)	3 (60.0)	2 (33.3)	–	2 (33.3)	4 (36.4)	1 (9.1)	5 (45.5)
	Febrile neutropenia	2 (40.0)	–	2 (40.0)	2 (33.3)	–	2 (33.3)	4 (36.4)	–	4 (36.4)
	Neutropenia	–	1 (20.0)	1 (20.0)	–	–	–	–	1 (9.1)	1 (9.1)
Gastrointestinal disorders		1 (20.0)	–	3 (60.0)	1 (16.7)	–	3 (50.0)	2 (18.2)	–	6 (54.5)
	Constipation	–	–	1 (20.0)	–	–	1 (16.7)	–	–	2 (18.2)
	Diarrhea	–	–	1 (20.0)	–	–	1 (16.7)	–	–	2 (18.2)
	Dyspepsia	–	–	1 (20.0)	–	–	–	–	–	1 (9.1)
	Nausea	1 (20.0)	–	3 (60.0)	1 (16.7)	–	3 (50.0)	2 (18.2)	–	6 (54.5)
	Stomatitis	–	–	1 (20.0)	–	–	–	–	–	1 (9.1)
	Vomiting	–	–	2 (40.0)	–	–	2 (33.3)	–	–	4 (36.4)
General disorders and administration site conditions		1 (20.0)	–	5 (100.0)	1 (16.7)	–	4 (66.7)	2 (18.2)	–	9 (81.8)
	Asthenia	1 (20.0)	–	5 (100.0)	1 (16.7)	–	4 (66.7)	2 (18.2)	–	9 (81.8)
	Pyrexia	–	–	–	–	–	1 (16.7)	–	–	1 (9.1)
Infections and infestations		–	–	–	–	–	1 (16.7)	–	–	1 (9.1)
	Gingivitis	–	–	–	–	–	1 (16.7)	–	–	1 (9.1)
	Neutropenic infection	–	–	–	–	–	1 (16.7)	–	–	1 (9.1)
Musculoskeletal and connective tissue disorders		–	–	1 (20.0)	–	–	1 (16.7)	–	–	2 (18.2)
	Muscle spasms	–	–	1 (20.0)	–	–	1 (16.7)	–	–	2 (18.2)
	Myalgia	–	–	1 (20.0)	–	–	–	–	–	1 (9.1)
Nervous system disorders		–	–	–	–	–	1 (16.7)	–	–	1 (9.1)
	Dysgeusia	–	–	–	–	–	1 (16.7)	–	–	1 (9.1)
Psychiatric disorders		–	–	–	–	–	1 (16.7)	–	–	1 (9.1)
	Confusional state	–	–	–	–	–	1 (16.7)	–	–	1 (9.1)
Skin and subcutaneous tissue disorders		–	–	1 (20.0)	–	–	3 (50.0)	–	–	4 (36.4)
	Alopecia	–	–	1 (20.0)	–	–	1 (16.7)	–	–	2 (18.2)
	Night sweats	–	–	–	–	–	1 (16.7)	–	–	1 (9.1)
	Onycholysis	–	–	–	–	–	1 (16.7)	–	–	1 (9.1)

*TESS* treatment emergent signs and symptoms

### Efficacy

Among the 11 patients treated, only 5 (45%) patients were evaluable for efficacy. Six (55%) patients were not evaluable because of premature discontinuation of study drug administration after cycle 1; DLT in 5/6 and treatment-unrelated toxicity for 1/6 patients. Among the 5 patients evaluable for efficacy, no objective response was observed. Only stable disease (SD) was reported as best overall response in 2 (40%) patients having both received 9 cycles, 1 at each DL.

### Pharmacokinetics and pharmacodynamics

**Fig. 1:**
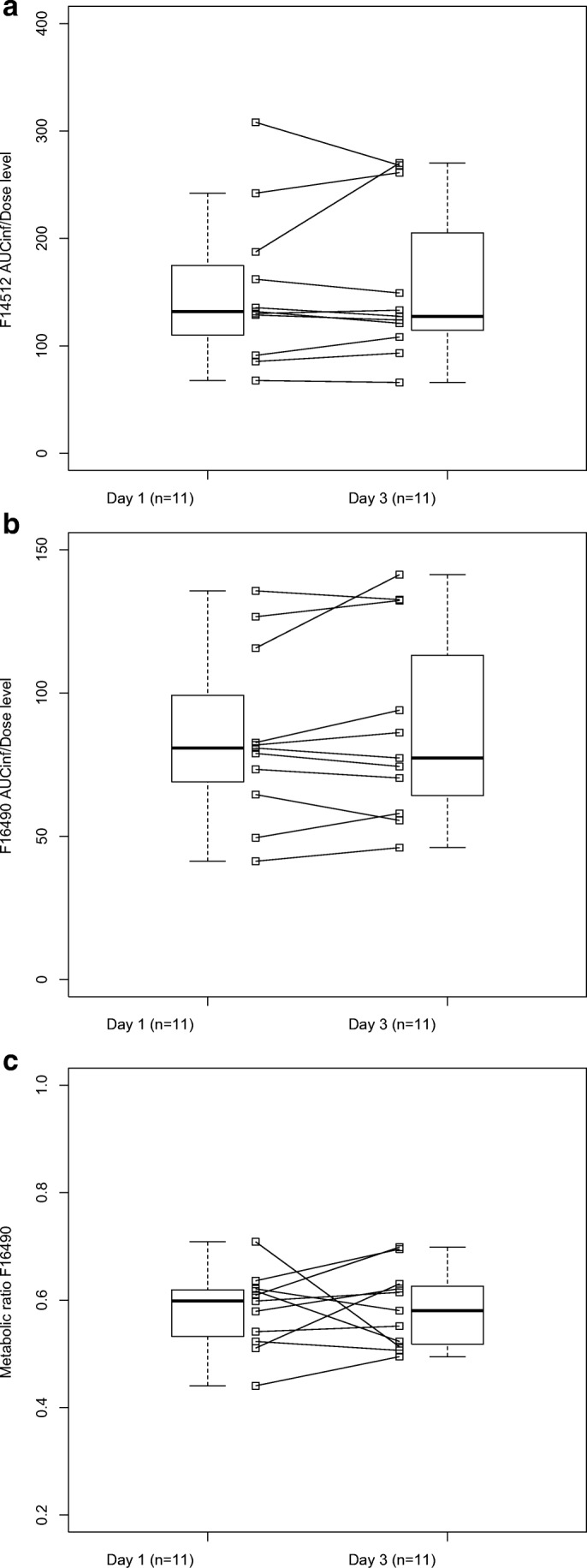
Comparison of F14512 AUC_inf_/dose level between day 1 and day 3 (**a**). Comparison of F16490 AUC_inf_/dose level between day 1 and day 3 (**b**). Comparison of F16490 metabolic ratio between day 1 and day 3 (**c**)

All 11 patients enrolled in the study were evaluable for PK and PD assessments. Two DLs, 5 mg/m^2^/day and 10 mg/m^2^/day, were tested. The PK profiles of F14512 and its metabolite F16490 were comparable between the 2 DLs. Concentrations of the BG metabolite were low and close to the lower limit of quantification. The inter-day reproducibility was evaluated for both metabolites and it is summarized in Table 4. No difference between Day 1 and Day 3 was observed. Because only 2 DLs were explored, no statistical analyses were performed to assess the dose proportional increase of AUC_inf_ or C_max_. However, F14512 and F16490 dose dependent PK parameters increased with DL. For inter-day reproducibility, parameters were compared using a pairwise t-test or a Wilcoxon test (for AUC_inf_/dose of F14512) and no significant associations were found (Fig. 1a). There was also no significant difference between metabolite F16490 AUC_inf_/dose (Fig. 1b) and metabolic ratio (Fig. 1c) on day 1 and day 3. CA-125 and HE 4 were quantified at the end of each cycle in all patients enrolled in the study. Due to the low number of samples per patient, no association can be tested between biomarkers and efficacy.

**Table 4:** Inter-day reproducibility of F14512 and its metabolite F16490

PK parameter	F14512 (n = 11) Mean [range]		F16490 (n = 11) Mean [range]	
	D1	D3	D1	D3
AUC_inf_/dose level	152 [67.7–308]	156 [65.9–270]	84.6 [41.3–136]	88.0 [46.0–141]
Cl_tot_ (L/h)	13.2 [6.17–22.4]	12.9 [5.92–23.1]	–	–
Metabolic ratio	–	–	0.580 [0.440–0.709]	0.584 [0.495–0.698]

## Discussion

Despite good initial responses to frontline treatment with debulking surgery and platinum-based chemotherapy, most patients with advanced ovarian cancer relapse and ultimately succumb from platinum-resistant disease. There are currently no effective therapeutic options for patients with PROC and therefore, there is an urgent need for novel therapies. Topo II inhibitors are active drugs in ovarian cancer and PLD is a standard of care both in combination with carboplatin and as monotherapy. However, response rates to single-agent PLD in PROC remain modest at 5–10% [16].

This report details the results of a phase 1 study of F14512 in patients with platinum-refractory or resistant ovarian cancer. An escalating dose of F14512 was to be administered, starting from the dose of 10 mg/m^2^/day. However, the first 3 patients treated at dose level 10 mg/m^2^/day experienced a DLT. Therefore, the DL below, which was 5 mg/m^2^/day, was tested. Among the first 3 treated patients at DL 5 mg/m^2^/day, no DLT was observed. Three more patients were treated and 2 of them experienced a DLT. Thus, both DLs of 5 mg/m^2^/day and 10 mg/m^2^/day were defined as MTD. Although grade 4 neutropenia was experienced by 90.9% of all treated patients and observed in 26/29 cycles administered, it is of note that only for 1 patient this neutropenia was reported as a serious drug-related AE.

At DL 10 mg/m^2^/day, hematological toxicity (grade 4 neutropenia) was observed in all patients and in 12/13 cycles administered. At DL 5 mg/m^2^/day, hematological toxicity (grade 4 neutropenia) was also observed in all patients and in 14/16 cycles administered. For non-hematological toxicity, at DL 10 mg/m^2^/day the most common AEs observed were asthenia, decreased appetite and nausea. Of these, 1 nausea, 1 asthenia and 1 decreased appetite occurring at cycle 1 were grade 3 (DLTs). At dose level 5 mg/m^2^/day, the most common non-hematological toxicities observed were asthenia and nausea. Of these, 2 nausea, 2 asthenia and 2 decreased appetite were grade 3. SAEs were reported in 3 patients at each DL and were hematological toxicities. Of these, 5 were DLTs; grade 3 febrile neutropenia was reported in 2 patients at each DL and grade 4 neutropenia lasting at least 7 days was reported in 1 patient at DL 10 mg/m^2^/day. One grade 2 neutropenic infection was reported as SAE at DL 5 mg/m^2^/day, occurring at cycle 9. It should be stressed that during the first cycle, G- and GM-CSF use was prohibited.

Among the 5 patients evaluable for efficacy, no response was observed. Only SD was reported as best overall response in 2 patients having both received 9 cycles, 1 at each DL.

The PK profiles of F14512 and its metabolite F16490 in patients with ovarian cancer were comparable between DLs. Difference of PK parameters between Day 1 and Day 3 of both compounds were not statistically significant. The small number of DLs and patients did not allow statistical analysis of the linearity between DLs. However, the results suggested that the dose proportionality was respected. No reliable PK/PD analysis can be performed. It is of note that the higher grade of neutropenia, grade 4, was observed in the patient with the highest exposure to the compound.

F14512 has demonstrated promising activity in preclinical studies of both solid and hematological malignancies, including ovarian cancer [4, 10, 11, 14]. The first-in-man phase I study of F14512 as a single-agent in patients with relapsed or refractory acute myeloid leukemia (AML) demonstrated both minimal extramedullary toxicity and promising antileukemic activity, with complete remissions in 36% of patients at first relapse [17]. Based on its favorable safety profile and its promising antileukemic activity, F14512 is in further clinical investigation in patients with AML and it is currently being tested in a phase I-II study in combination with cytarabine.

In conclusion, F14512 infusion over 3 h administered for 3 consecutive days every 3 weeks at DLs of 10 mg/m^2^/day and 5 mg/m^2^/day, in platinum-resistant or refractory ovarian cancer, led to high incidence of grade 4 neutropenia reported in 90.9% of patients and in 89.7% of cycles administered. This grade 4 neutropenia led to a high rate of complications; febrile neutropenia was observed in 2 patients at each DL and neutropenic infection in 1 patient at DL 5 mg/m^2^/day. Therefore, it was decided to stop the study and a DL below 5 mg/m^2^/day was not tested. This was because a DL below 5 mg/m^2^/day did not allow reaching the concentration needed for the pharmacological activity of the drug. Given that predictable hematological toxicity has prevented adequate dose-escalation and the encouraging clinical efficacy demonstrated in AML, the addition of G-CSF to enable dose-escalation and full evaluation in ovarian cancer should be considered in case of further development.

## Electronic supplementary material

ESM 1 (DOCX 20 kb)
