# Supplementary material for: Phase I dose-escalation study of F14512, a polyamine-vectorized topoisomerase II inhibitor, in patients with platinum-refractory or resistant ovarian cancer
Source: Invest New Drugs. 2018 Dec 14;37(4):693–701. doi: 10.1007/s10637-018-0688-4 (PMC6647401; doi:10.1007/s10637-018-0688-4)
Supplement: Supplementary file 1 — (DOCX 20 kb) [file 10637_2018_688_MOESM1_ESM.docx]

**Title: Phase I dose-escalation study of F14512, a polyamine-vectorized topoisomerase II inhibitor, in patients with platinum-refractory or resistant ovarian cancer**

**Journal:** Investigational New Drugs

**Authors:** Alexandra Leary, Christophe Le Tourneau, Andrea Varga, Marie-Paule Sablin, Carlos Gomez-Roca, Nicolas Guilbaud, Aurelie Petain, Mariya Pavlyuk, Jean-Pierre Delord

**Corresponding author:** Professor Jean-Pierre Delord, MD, PhD; Institut Universitaire du cancer de Toulouse Oncopole - IUCT Oncopole, 1 avenue Irène Joliot-Curie, 31100 Toulouse, France; Tel: +33 53115 5101,
Fax: +33 53115 5002 ; Email : [Delord.Jean-Pierre@iuct-oncopole.fr](mailto:Delord.Jean-Pierre@iuct-oncopole.fr)

**Online resource – Online only**

**Methods**

**Patients and study design**

Patient eligibility criteria included: measurable (according to RECIST version 1.1) or CA-125 assessable (≥ 2 times upper limit of the reference range at least twice within the 2 weeks before study entry) disease according to GCIG criteria or evaluable disease (defined as histologically proven recurrence or evidence of ascites or pleural effusion), minimal interval of 3 weeks between the completion of prior chemotherapy and study entry, prior radiotherapy was allowed provided that a minimum of 3 weeks had elapsed before study entry, age between 18 and 75 years, WHO performance status ≤1, anticipated life expectancy ≥12 weeks, adequate hematological function defined as ANC ≥ 1.5 x 109/L, platelet count ≥ 100 x 109/L and hemoglobin ≥ 9 g/dL, adequate liver function tests defined as total bilirubin ≤ Upper Limit of Normal (ULN), AST and ALT ≤ 3 x ULN, adequate renal function defined as serum creatinine ≤ 1.5 x ULN, and left ventricular ejection fraction ≥ 45% (MUGA scan or bi-dimensional echography). Patients with prior treatment by more than two chemotherapy regimens, clinical symptoms of central nervous system involvement, current active infection, any concurrent and/or uncontrolled medical disorder, HIV, HTLV1, Hepatitis B or C positivity, history of another malignancy within the past five years (except basal cell carcinoma of the skin or carcinoma in situ of the cervix, and surgically-treated-only or lobular carcinoma in situ of the breast diagnosed more than 5 years ago), active heart disease including myocardial infarction within the previous 6 months, symptomatic coronary artery disease, arrhythmia not controlled by medication or uncontrolled congestive heart failure, history of cardiac conduction abnormality grade ≥ 2 (NCI CTCAE V4.0), major surgery within 28 days prior to study entry, concurrent treatment with any other anti-cancer therapy, participation in another trial of an investigational agent within 30 days before study entry, hypersensitivity to the study drug or to drugs with similar chemical structures, concurrent treatment with inhibitors of ornithine decarboxylase or polyamine analogues, were not eligible.

**Dose-limiting toxicities (DLTs)**

DLTs were defined as one of the following drug-related AEs occurring during the first cycle: grade 4 neutropenia ≥ 7 days, grade 4 thrombocytopenia, or grade 3 thrombocytopenia requiring transfusion and/or associated with bleeding, grade 3 or 4 neutropenia concomitant with grade ≥ 3 infection or febrile neutropenia, any grade ≥ 3 gastrointestinal toxicity including grade ≥ 3 nausea, vomiting or diarrhea if persistent despite optimal antiemetic or anti-diarrheal treatment, any other drug-related grade ≥ 3 toxicity, any drug-related AE that required a delay of ≥ 2 weeks of the administration of cycle 2.

**Safety assessment**

Safety was assessed by physical examination on days 1, 2 and 3 during each cycle. Complete blood cell counts were performed on days 4, 6, 8, 10, 12 and 15 of the first cycle, on day 1 of each subsequent cycle and then on a weekly basis thereafter. Serum chemistry was performed weekly during the first cycle and at the end of each cycle thereafter except serum magnesium and potassium which were measured twice weekly during the first week of each cycle and then, weekly for the following weeks. ECGs were performed at baseline and on day 1 of each cycle.

**Pharmacokinetics (PK)**

Plasma PK was studied after the administrations on days 1 and 3 of cycle 1 (pre-dose, T0+1.5 h, T0+3 h, T0+3.5 h, T0+4 h, T0+6 h T0+12 h, T0+24 h) and day 1 of cycle 2 (pre-dose, T0+3.5 h, T0+8 h, T0+24 h). F14512, its metabolite F16490 and the metabolite BG (an additional metabolite peak found in human during exploratory metabolite identification analysis and not retrieved in animal species) were assayed in plasma using liquid chromatography coupled with tandem mass spectrometry (LC/MS/MS).

**Statistics**

Continuous data were summarized with frequency, median, range, mean, standard deviation and standard error if relevant. Categorical data were presented as frequencies and percentages. 95% confidence intervals (CI) were calculated following the exact method. PK parameters were compared for reproducibility between day 1 and day 3 of cycle 1 for F14512 and its metabolite F16490 using a pairwise t-test or a Wilcoxon test according to the normality of their distribution. Dose-proportionality of PK parameters for F14512 and its metabolite F16490 was investigated by graphical display only. A p value <0.05 was considered statistically significant. Data were analyzed using the SAS® system software version 9.3 for Windows® (Statistical Analysis System, Cary, NC, USA).
